# Mrp Antiporters Have Important Roles in Diverse Bacteria and Archaea

**DOI:** 10.3389/fmicb.2017.02325

**Published:** 2017-11-23

**Authors:** Masahiro Ito, Masato Morino, Terry A. Krulwich

**Affiliations:** ^1^Graduate School of Life Sciences, Toyo University, Gunma, Japan; ^2^Bio-Nano Electronics Research Center, Toyo University, Kawagoe, Japan; ^3^Department of Pharmacological Sciences, Icahn School of Medicine at Mount Sinai, New York, NY, United States

**Keywords:** alkaliphile, cation/proton antiporter, Mrp, complex I, multi-subunit antiporter, *Bacillus*, *Thermomicrobium*

## Abstract

Mrp (Multiple resistance and pH) antiporter was identified as a gene complementing an alkaline-sensitive mutant strain of alkaliphilic *Bacillus halodurans* C-125 in 1990. At that time, there was no example of a multi-subunit type Na^+^/H^+^ antiporter comprising six or seven hydrophobic proteins, and it was newly designated as the monovalent cation: proton antiporter-3 (CPA3) family in the classification of transporters. The Mrp antiporter is broadly distributed among bacteria and archaea, not only in alkaliphiles. Generally, all Mrp subunits, *mrpA–G*, are required for enzymatic activity. Two exceptions are Mrp from the archaea *Methanosarcina acetivorans* and the eubacteria *Natranaerobius thermophilus*, which are reported to sustain Na^+^/H^+^ antiport activity with the MrpA subunit alone. Two large subunits of the Mrp antiporter, MrpA and MrpD, are homologous to membrane-embedded subunits of the respiratory chain complex I, NuoL, NuoM, and NuoN, and the small subunit MrpC has homology with NuoK. The functions of the Mrp antiporter include sodium tolerance and pH homeostasis in an alkaline environment, nitrogen fixation in *Schizolobium meliloti*, bile salt tolerance in *Bacillus subtilis* and *Vibrio cholerae*, arsenic oxidation in *Agrobacterium tumefaciens*, pathogenesis in *Pseudomonas aeruginosa* and *Staphylococcus aureus*, and the conversion of energy involved in metabolism and hydrogen production in archaea. In addition, some Mrp antiporters transport K^+^ and Ca^2+^ instead of Na^+^, depending on the environmental conditions. Recently, the molecular structure of the respiratory chain complex I has been elucidated by others, and details of the mechanism by which it transports protons are being clarified. Based on this, several hypotheses concerning the substrate transport mechanism in the Mrp antiporter have been proposed. The MrpA and MrpD subunits, which are homologous to the proton transport subunit of complex I, are involved in the transport of protons and their coupling cations. Herein, we outline other recent findings on the Mrp antiporter.

## Diversity of Na^+^/H^+^ Antiporters

The Na^+^/H^+^ antiporter is a secondary active transporter that utilizes the proton motive force to efflux intracellular sodium ions (Padan et al., 2005; Krulwich et al., 2011; Fuster and Alexander, 2014; Padan and Landau, 2016). It is a widely distributed membrane protein, and studies of it are being conducted in eukaryotic-derived NHE families as well as bacterial-derived NhaA families (Wakabayashi et al., 1997; Orlowski and Grinstein, 2004; Padan, 2014; Padan and Landau, 2016). The main physiological roles of the Na^+^/H^+^ antiporter are intracellular pH homeostasis and Na^+^ efflux. Na^+^ efflux by the Na^+^/H^+^ antiporter plays a critical role for sodium circulation inside and outside the cell because many bacteria, including marine bacteria, utilize both the proton motive force and sodium motive force (Zilberstein et al., 1982; Padan and Schuldiner, 1994; Vimont and Berche, 2000; Dibrov, 2005; Krulwich et al., 2011).

NHE, a mammalian Na^+^/H^+^ exchanger, is a group of 12-transmembrane membranes protein with multiple isoforms (Wakabayashi et al., 1997; Orlowski and Grinstein, 2011; Padan and Landau, 2016). The antiporters designated as NHE1–NHE5 are localized in the cell plasma membrane, while NHE6–NHE9 are present in the membranes of intracellular organelles (Ohgaki et al., 2011; Fuster and Alexander, 2014). Furthermore, NHE has a hydrophilic domain on the carboxyl-terminal side exposed to the cytoplasm. The interaction between this hydrophilic domain and calcineurin, which is a Ca^2+^-dependent serine/threonine protein phosphatase, is reportedly involved in intracellular pH homeostasis and is crucial for NHE ion transport activity (Wakabayashi et al., 1997; Pang et al., 2001; Hisamitsu et al., 2012). In NHE 1, it has been reported that enzymatic activity is activated in response to various stimuli including hormones, growth factors, and mechanical stress (Wakabayashi et al., 1997; Hisamitsu et al., 2012). Mammalian NHE has high homology with the bacterial NhaP family, while it has low homology with the bacterial NhaA family, a member of the bacterial Na^+^/H^+^ antiporter family (Waditee et al., 2001; Resch et al., 2011; Padan and Landau, 2016). In addition, the NhaP antiporter family has been shown to have a large hydrophilic domain at its carboxy-terminal side like NHE (Waditee et al., 2001; Mourin et al., 2017).

In general, bacteria have multiple Na^+^/H^+^ antiporters that are thought to exert appropriate responses to the ambient conditions of the growth environment and its associated stresses (Padan et al., 2005; Krulwich et al., 2011; Padan, 2014; Preiss et al., 2015). For example, *Escherichia coli* has three major Na^+^/H^+^ antiporters designated as NhaA, NhaB, and ChaA. It has been shown that NhaA is expressed as a response to the stress associated with alkaline pH and sodium ions in *E. coli* (Padan et al., 2001, 2005; Padan, 2014; Padan and Landau, 2016). Furthermore, NhaA is activated in alkaline pH while NhaB retains activity only in neutral pH; therefore, NhaA is thought to play a central role in the adaptation of *E. coli* to an alkaline environment. In addition to these findings, it has been shown that ChaA is a Ca^2+^/H^+^ antiporter and MdfA is a multidrug/proton antiporter that retains Na^+^/H^+^ antiport activity (Ivey et al., 1993; Shijuku et al., 2002; Lewinson et al., 2003).

## Discovery of the Mrp Gene Cluster

**FIGURE 1:**
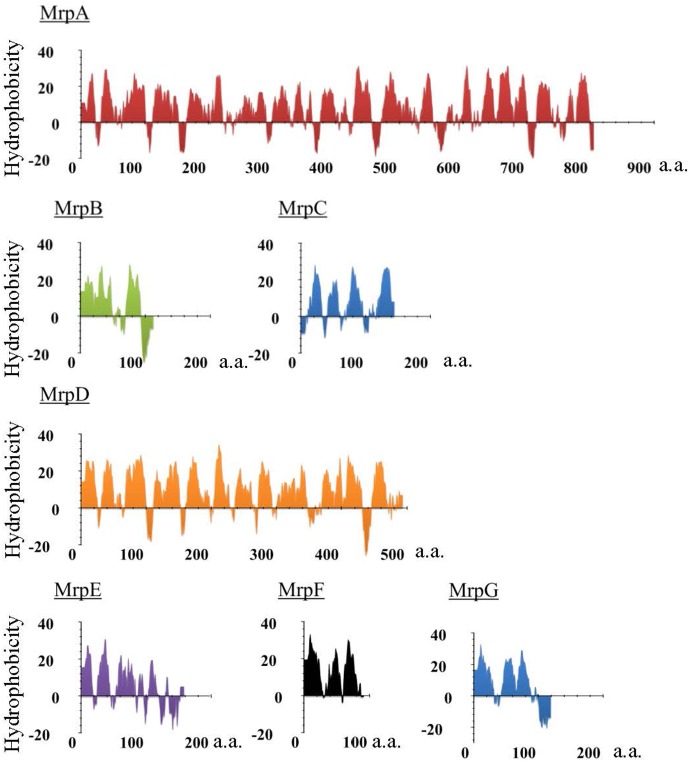
Hydropathy profile of the Mrp subunits derived from alkaliphilic *B. pseudofirmus* OF4. The hydropathy profile of the Mrp subunits derived from *B. pseudofirmus* OF4 was predicted using Kyte and Doolittle method. The vertical axis represents the degree of hydrophobicity, and the horizontal axis represents the number of amino acids (a.a.).

Mrp was first discovered in work on an alkaline-sensitive strain of alkaliphilic *Bacillus halodurans* C-125 in Kudo et al. (1990). It was found that the gene cluster encoded a Na^+^/H^+^ antiporter (Hamamoto et al., 1994). The *mrp* gene cluster of *B. halodurans* C-125 comprises seven *mrp* genes (*mrpABCDEFG*), and the expressed proteins are predicted, from the amino acid sequence, to all be membrane proteins (**Figure 1** and **Table 1**). The Mrp antiporter has been suggested to function as a complex of multiple membrane proteins (Kajiyama et al., 2007; Morino et al., 2008). Apart from the maintenance of cytoplasmic pH, the Mrp complex has various other physiological roles in different species, such as bile acid resistance in *Bacillus subtilis* and *Vibrio cholera* (Ito et al., 1999; Dzioba-Winogrodzki et al., 2009), Na^+^ homeostasis/tolerance in *B. subtilis* (Ito et al., 1999; Kosono et al., 1999; Ito et al., 2000), sporulation in *B. subtilis* (Kosono et al., 2000), plant infection in *Sinorhizobium meliloti* (Putnoky et al., 1998), pathogenesis in *Pseudomonas aeruginosa* (Kosono et al., 2005) and arsenic resistance in *Agrobacterium tumefaciens* (Kashyap et al., 2006).

**Table 1:** Molecular weight of each Mrp subunit derived from the *B. pseudofirmus* OF4 strain and the estimated number of transmembrane regions.

Protein	Estimated molecular weight (kDa)	Estimated transmembrane segment number^a^
MrpA	89.4	19–21
MrpB	15.8	4
MrpC	12.2	3
MrpD	54.4	14
MrpE	18.4	2–3
MrpF	10.0	3
MrpG	13.1	3
Total	213.4	48–51


*^a^The estimated transmembrane segment number was estimated by using the TMHMM (http://www.cbs.dtu.dk/services/TMHMM/) and HMMTOP (http://www.enzim.hu/hmmtop/) programs, which are transmembrane segment prediction software, for the amino acid sequence of each subunit. MrpA was predicted as a 21-transmembrane proten by TMMTOP and TMHMM, but as a 19-transmembrane proteins by ConPred II. MrpE was predicted as a two-transmembrane protein by TMMTOP and TMHMM, but as a three-transmembrane one by ConPred II.*

## Phylogenetic Analysis of the Mrp Gene Cluster

**FIGURE 2:**
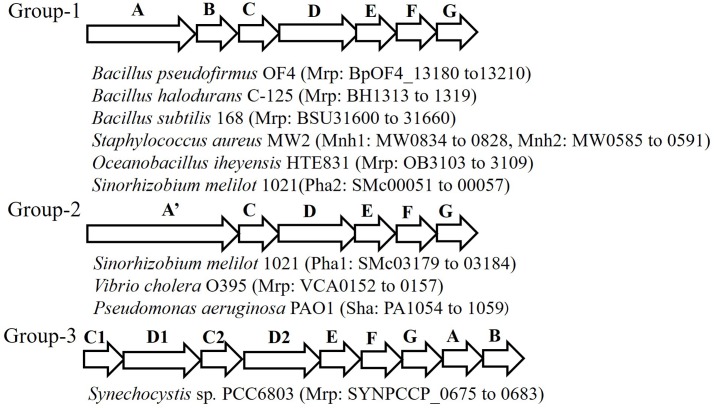
Schematic of the diversity of the *mrp* gene cluster in several bacteria. This figure depicts the genetic structure of the *mrp* gene cluster of each bacterium. The locus tag numbers of each Mrp subunit gene are listed after the bacterial name. *mrpA’* is a gene involving the fusion of *mrpA* and *mrpB*.

The Mrp antiporter has been found in alkaliphilic bacteria as well as in many other bacteria and archaea. Genome analyses in a wide range of microorganisms clarified that the structure of the *mrp* gene cluster is diverse (**Figure 2**) (Swartz et al., 2005; Krulwich et al., 2009). Because of its distinctive properties, Mrp antiporter systems have been classified in their own category, cation: proton antiporter-3 (CPA3), in the transporter classification system (Saier et al., 2009, 2016). So far, the *mrp* gene cluster has been classified into three groups. Group 1 antiporters are composed of seven *mrp* genes, and it is found in many *Bacillus* spp. and in *Staphylococcus aureus*. Group 2 has a *mrp* gene cluster *(mrpA’CDEFG*) of six genes. This group belongs to bacteria such as *Pseudomonas aeruginosa* and *Vibrio cholerae*, in which it appears that the *mrpA* gene is fused with the *mrpB* gene encoding a fusion protein (Kosono et al., 2005; Swartz et al., 2005; Dzioba-Winogrodzki et al., 2009). *Sinorhizobium meliloti* has two sets of *mrp* (alias *pha*) gene clusters, one belongs to Group 1 (Pha2) and the other belongs to Group 2 (Pha1) (Putnoky et al., 1998; Yamaguchi et al., 2009). The *mrp* gene cluster belonging to Group 3 has each subunit, but the gene order is irregular. For example, the *mrp* of cyanobacteria has two *mrpB* genes, and the gene sequence in the gene cluster is as follows: *mrpCDCDEFGBB* (Waditee et al., 2001).

*Staphylococcus aureus* has been shown to have two sets of group 1 type *mrp* (alias *mnh*) gene clusters, *mnh1* and *mnh2. mnh1* has been found to encode the Na^+^/H^+^ antiporter; however, the function of the product encoded by *mnh2* remains unknown (Swartz et al., 2007). Similarly, genomic analyses have revealed that alkaliphilic *Bacillus clausii* and the marine bacterium *Oceanobacillus iheyensis* have two sets of *mrp* gene clusters (Krulwich and Ito, 2013). However, there are no reported examples of the physiological and functional differences between them. In addition, analysis of many microbial genomes has revealed three *mrp* gene clusters. For example, *Microbacterium* sp. TS-1 has three sets of Mrp gene clusters, two of them (locus tags, MTS1_01879-01874 and MTS1_02182-02187) belong to Group 2 and the third one (locus tags, MTS1_02374-02382; *mrpFGBCDDAE*) belongs to Group 3 (Fujinami et al., 2013) and hyperthermophilic archaeon, *Thermococcus onnurineus* NA1 has three sets of Mrp gene clusters, all of which (locus tags; TON_0272-0266, TON_1574-1580, TON_1025-1031) belong to Group 1 (Lim et al., 2010).

The *mrp* gene cluster of anaerobic bacteria has a gene structure that is markedly different from that of aerobic bacterial-derived *mrp* gene clusters. For example, the *mrp* gene cluster of *Natranaerobius thermophilus* retains three overlapping *mrpB* genes (Mesbah et al., 2009). In addition, in the *mrp* gene cluster derived from *Synechocystis* sp. PCC 6803, duplication of the *mrpD* and *mrpC* genes as well as the *mrpB* gene is observed (Krulwich et al., 2009). Similar gene arrangements have been reported in other cyanobacteria (Fukaya et al., 2009).

## Relationship Between Mrp Antiporter and Respiratory Chain Complex I

**FIGURE 3:**
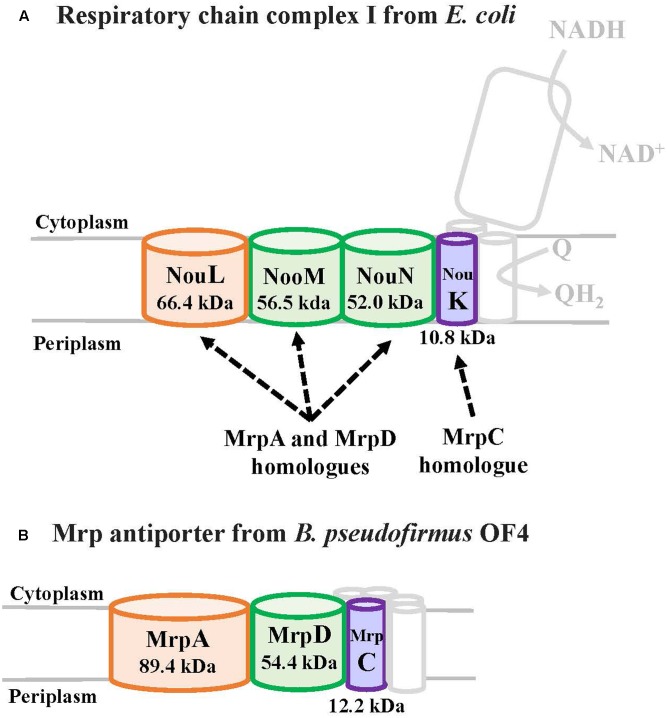
The *E. coli* respiratory chain complex I subunit with homology to the Mrp subunit. **(A)** Schematic diagram of respiratory chain complex I. The NuoL, NuoM, and NuoN subunits of the *E. coli* respiratory chain complex I have homology with the MrpA and MrpD subunits of the Mrp antiporter, and the NuoK subunits also have partial homology with the MrpC subunit (Mathiesen and Hägerhäll, 2003). **(B)** Schematic diagram of Mrp antiporter.

**FIGURE 4:**
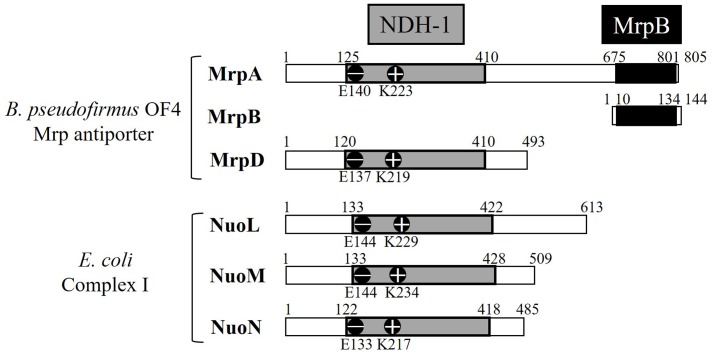
Common regions preserved between the *B. pseudofirmus* OF4 Mrp antiporter and the *E. coli* complex I subunit. MrpA, MrpD, NuoL, NuoM, and NuoN proteins having homology to each other are described. The NDH-1 domains conserved among them are represented in gray. The glutamic acid residue (–) and the lysine residue (+) conserved in the NDH-1 region are shown in the schematic. The C-terminal region of MrpA, in black, has been found to be systematically related to the MrpB protein by PSI-Blast analysis (Mathiesen and Hägerhäll, 2003).

MrpA and MrpD subunits have homology with the respiratory chain complex I subunit (**Figures 3**, **4**) (Mathiesen and Hägerhäll, 2003; Moparthi and Hägerhäll, 2011; Moparthi et al., 2014). The respiratory chain complex I is a protein complex belonging to the electron transport system, which oxidizes NADH supplied from the TCA cycle, among other sources. It reduces quinone and effluxes protons from the cell. The NuoL, NuoM, and NuoN subunits, which are subunits of the respiratory chain complex I in *E. coli*, have been analyzed because of homology with the Mrp antiporter subunit (Nakamaru-Ogiso et al., 2003a,b, 2010; Torres-Bacete et al., 2007; Ohnishi et al., 2010; Torres-Bacete et al., 2011; Sperling et al., 2016; Morino et al., 2017). These three Nuo subunits have highly conserved glutamic acid residues and lysine residues (**Figure 4**), which have been suggested to be the core of the proton transport pathway, based on the crystal structure of *E. coli* (Baranova et al., 2007; Efremov and Sazanov, 2011; Sazanov, 2014). In MrpA and MrpD subunits, these charged residues are highly conserved, and it has been reported that glutamate residues are also conserved at the same position in *B. subtilis* and *B. pseudofirmus* OF4. Mrp antiporters have been shown to be essential for antiport activity in various settings (Kosono et al., 2005; Kajiyama et al., 2009; Morino et al., 2010).

The crystal structure of the *E. coli* respiratory chain complex I revealed that the NuoL subunit had a long helical chain at its carboxy terminus (Efremov and Sazanov, 2011; Sazanov, 2014). Analysis of a long-chain, helix-deficient strain of the NuoL subunit in *E. coli* respiratory chain complex I indicated that this helix is indispensable for proton transport, complex formation, and NADH oxidation (Ohnishi et al., 2010; Efremov and Sazanov, 2011; Torres-Bacete et al., 2011; Sazanov, 2014). This suggested that it functions as a “piston” that couples oxidation and quinone reduction to proton transport. The MrpA subunit has an additional transmembrane region at the carboxy terminus similar to the NuoL subunit. In addition, part of the MrpA carboxy terminus has high sequence homology with MrpB, as shown by PSI-Blast analysis; it is speculated that it is a characteristic region only of the Mrp antiporter (Krulwich et al., 2009). Recently, it was reported that the MrpA carboxy-terminal region of *B. pseudofirmus* OF4 has indispensable roles in antiport function (Morino et al., 2017).

## Features of the Mrp Antiporter from Alkaliphilic *Bacillus pseudofirmus* OF4

Within the Mrp antiporter family, the *B. pseudofirmus* OF4-derived Mrp antiporter (Bp–Mrp) has undergone advanced functional and structural analyses that has revealed: (1) formation of the complex and role of each subunit; (2) identification of amino acid residues with important structural and functional roles, as determined by site-specific functional analysis; (3) analysis of the specific C-terminal region of MrpA; and (4) purification and reconstitution of the Bp–Mrp antiporter.

### Formation of Bp–Mrp Complex and the Role of Each Mrp Subunit

Bp–Mrp was estimated to form a membrane protein complex expressed from seven *mrp* genes. Bp–Mrp expressed in *E. coli* was separated by Blue native PAGE (BN-PAGE); subsequently, each Mrp subunit was detected by Western blotting to investigate whether the Mrp antiporter successfully formed a complex (Morino et al., 2008). The results confirmed formation of a Mrp complex (220 kDa), estimated to be a monomer consisting of all subunits, as well as a MrpABCDEFG complex (400 kDa), estimated to be a dimer. A MrpABCD subcomplex comprising MrpA, B, C, and D subunits was also detected; this subcomplex was shown not to be catalytically active (Morino et al., 2008).

Mutants were also constructed, each with the deletion of a single *mrp*, to enable investigation of the role of each Mrp subunit in complex formation (Morino et al., 2008). The results showed that, in the membrane fraction of the *mrpD* deletion mutant, no other Mrp subunits were detected. On the other hand, Mrp subunits other than MrpE could be detected in the membrane of the *mrpE*-deficient mutant. From BN-PAGE analysis, it was confirmed that the Mrp subunits other than MrpE form a complex in the *mrpE* deletion mutant. These results suggested that the MrpD subunit is important in the formation of the Bp–Mrp complex. It may have a role as a scaffold when other Mrp subunits are expressed in the cell membrane. By contrast, the MrpE subunit appears to be incorporated in the final step of complex formation and possibly plays an important role in ensuring that the Mrp complex can exert its full activity. However, in *B. subtilis*, it was reported that MrpE is dispensable for ion transport activity (Yoshinaka et al., 2003; Morino et al., 2008).

### Site-Directed Amino Acid Substitution Mutagenesis and Identification of Residues in the Bp–Mrp Antiporter Important for Ion Transport

**FIGURE 5:**
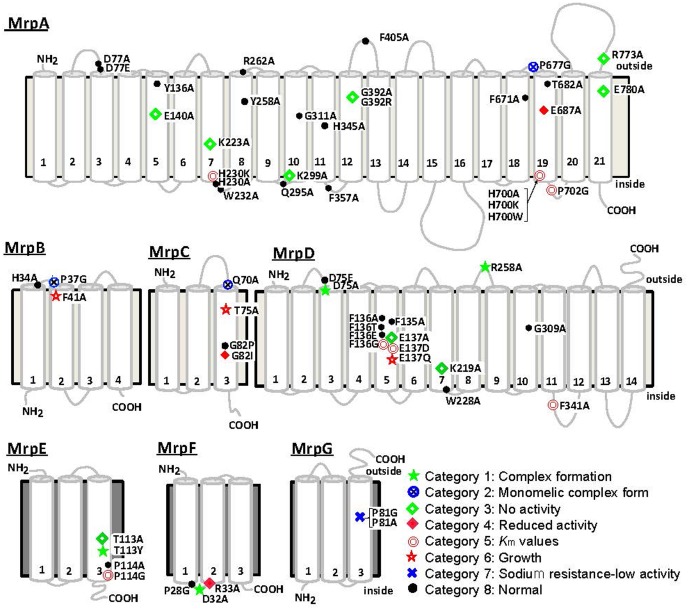
Transmembrane topology of Bp–Mrp proteins and positions of mutations. Transmembrane segments predicted by ConPred II, HMMTOP, and TMHMM (available online) were used in the analyses of the secondary structure predictions for each Mrp subunit. 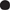, positions at which the mutations complemented an antiporter-deficient *E. coli* KNabc transformant and exhibited normal Na^+^/H^+^ antiport activity; 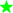, mutations that affected the level of Mrp proteins in the membrane; 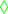, mutations that led to loss of the Na^+^/H^+^ antiport activity and loss of Na^+^ tolerance; 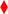, mutations that decreased the Na^+^/H^+^ antiport activity, without an effect on growth of the *E. coli* transformant; 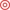, mutations that affected the *K*_m_ values of Mrp-dependent antiport activity; 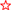, mutations that affected transformant cell growth; 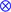, mutations that affected Mrp complex formation; 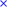, the two mutants in MrpG-P81 that had a unique phenotype.

The Bp–Mrp antiporter was studied to identify amino acid residues within it that are important for ion transport and Mrp complex formation. Site-specific mutations were introduced at amino acid residues conserved between Mrp homologs. In MrpA and MrpD subunits, mutations were also introduced at amino acid residues conserved among the NuoL, NuoM, and NuoN subunits of the homologous *E. coli* respiratory chain complex I (Morino et al., 2010). The mutants were expressed in the *E. coli* KNabc strain, in which three major Na^+^/H^+^ antiporter genes (*nhaA*, *nhaB*, and *chaA*) are deleted; subsequently, the mutants were tested for sodium sensitivity, antiport activity, and their complex formation ability. Each amino acid substitution mutant could be classified into one of eight categories from each phenotype. **Figure 5** shows a summary of the phenotype at each mutation site (Morino et al., 2010, 2017). Mutants classified into categories 1 and 2 have been shown to affect Mrp complex formation. Mutants classified into categories 3–7 were confirmed to undergo complex formation but resulted in a decrease in Na^+^/H^+^ antiport activity and a decrease in the sodium-sensitive complementary activity of *E. coli* KNabc.

In category 1, MrpD-D75A, MrpD-R258A, MrpE-T113Y, and MrpF-D32A were studied, and their Na^+^/H^+^ antiport activity was found to be completely lost, with no Mrp complex detected.

In category 2, MrpA-P677G, MrpB-P37G, and MrpC-Q70A mutations were associated with the retention of Na^+^/H^+^ antiport activity but failure to show formation of the Mrp complex monomer in BN-PAGE analysis. These mutations were assumed to destabilize the interaction between the MrpABCD subcomplex and each of the MrpE, MrpF, and MrpG subunits.

MrpA-E140A, MrpA-K223A, MrpA-K299A, MrpA-G392R, MrpA-R773A, MrpA-E780A, MrpD-E137A, MrpD-K219A, and MrpE-T113A, which are classified into category 3, retained the Mrp complex but Na^+^/H^+^ antiport activity was completely lost.

MrpC-G82I and MrpF-R33A, classified into category 4, exhibited Na^+^/H^+^ antiport activity that was decreased by approximately 70% compared with wild-type activity.

In category 5, the apparent *K*_m_ for Na^+^ of Na^+^/H^+^ antiport activity increased in MrpA-H230K, MrpA-H700A, MrpA-H700K, MrpA-H700W, MrpA-P702G, MrpD-F136G, MrpD-E137D, MrpD-F341A, and MrpE-P114G. Because MrpA-H230, MrpA-H700, MrpA-P702, and MrpD-F136 are adjacent to charged residues essential for activity (MrpA-K223, MrpA-E687, MrpD-E137), along with these charged residues, it is assumed that they are involved in ion transport along with these chargeable residues. Although the functional roles of MrpD-F341 and MrpE-P114 are unknown, it is inferred that the low-molecular-weight subunit MrpE may also be involved in ion transport.

MrpB-F41A and MrpC-T75A, classified into category 6, retained normal Na^+^/H^+^ antiport activity but could not completely complement the sodium sensitivity in *E. coli* KNabc.

Na^+^/H^+^ antiport activity was completely inactivated in MrpG-P81A, classified into category 7. Surprisingly, however, the sodium sensitivity of *E. coli* KNabc could be complemented similarly to that of the wild type (see below).

The amino acid substitution mutants that showed the same phenotype as the wild type were designated into category 8.

In MrpA-E140, MrpA-K223, MrpD-E137A, and MrpD-K219A, there was conservation of not only the MrpA and MrpD subunits but also the respiratory chain complex I subunit. They are extremely important for Na^+^/H^+^ antiport activity, and it was speculated from the complex I crystal structure that the respiratory chain complex I also participates in ion transport. In addition, MrpG-P81A in category 7 did not retain Na^+^/H^+^ antiport activity, but it was able to complement the sodium sensitivity of the *E. coli* KNabc. This suggested that the Mrp antiporter of MrpG-P81A has Na^+^ efflux capacity coupled with the transport of ions other than protons. For example, membrane potential-driven sodium ion excretion may occur concomitantly with the transport of anions. However, the phenotype of MrpG-P81A, including the possibility of having other transporting substrates, is only a hypothesis at this point; therefore, more detailed analyses are needed.

### Functional Analysis of the Carboxyl-Terminal Region of the MrpA Subunit of the Bp–Mrp Antiporter

The C-terminal region of MrpA, which has similarity to the MrpB subunit, is conserved. This region of MrpA is not preserved in the respiratory chain complex I subunit; therefore, it is predicted to have unique functions and roles in the Mrp antiporter. Site-specific mutations involving substitutions at highly conserved amino acid residues located in the C-terminal region of Bp–MrpA were introduced (Morino et al., 2017). Two glutamic acid residues are conserved in the C-terminal region of MrpA, as has been reported by Kosono et al. (2006) using *B. subtilis* Mrp. In the Bp–Mrp antiporter, these acidic residues (MrpA-E687 and MrpA-E778) are also essential for Na^+^/H^+^ antiport activity. In addition, MrpA-P683G retained normal Na^+^/H^+^ antiport activity; however, the monomeric MrpABCDEFG complex could not be detected by BN-PAGE analysis. The fact that the same phenotype is also found in MrpB-P37G and MrpC-Q70A suggested that the C-terminal region of MrpA is a region through which interactions with low-molecular-weight Mrp subunits, MrpB and MrpC, occur. In addition, it was observed that Na^+^/H^+^ antiport activity decreased in MrpA-P702G and MrpA-R773A mutants, suggesting that the C-terminal region of MrpA has an important function in ion transport.

The above results also showed that the C-terminal region of MrpA has important functions not only in ion transport but also in interactions between subunits. Furthermore, the C-terminal region of MrpA is a region unique to the Mrp antiporter and is suggested to be involved in Na^+^/H^+^ antiport activity.

### Purification and Reconstitution of the Bp–Mrp Antiporter

Reports have been published on structural analyses of various protein complexes by techniques such as single-particle analysis by the observation of high-purity samples under an electron microscope. For example, in the respiratory chain complex I, an L-shaped structure has been observed under an electron microscope (Holt et al., 2003). The structure of very large macromolecules, such as the H-ring, which is a component of the basal body of bacterial flagella, has also been clarified by microscopic observation (Terashima et al., 2010). High-purity samples of target proteins and complexes thereof are indispensable for such advanced structural analysis. As such, purification of the Mrp antiporter derived from *B. pseudofirmus* OF4 was investigated. The Mrp antiporter expressed in *E. coli* was purified by TALON resin and reconstituted into an artificial lipid membrane for further confirmation of its Na^+^/H^+^ antiport activity. In the reconstituted membrane, the proton motive force required for Mrp antiporter activation was generated by F_o_F_1_-ATPase derived from *Bacillus* sp. PS3, which was simultaneously reconstituted. This report is the first to describe the successful reconstitution of purified bacterial-derived Mrp antiporter into proteoliposomes retaining Na^+^/H^+^ antiport activity (Morino et al., 2014).

## Mrp Antiporters from Gram-Positive Bacteria Other than Alkaliphilic *Bacillus* spp.

It was shown that the *mrp* (alias *sha*) gene cluster of *B. subtilis* encodes a Na^+^/H^+^ antiporter and plays a major role in the mechanism of sodium tolerance of *B. subtilis* (Ito et al., 1999; Kosono et al., 1999). Various *mrp*-deficient strains have been produced in *B. subtilis*, and it has been reported from their analysis that the *mrpF* gene contributes to bile acid tolerance (Ito et al., 1999). Furthermore, it has been reported that sodium efflux capacity is retained in a *mrpE* gene-deficient strain (Yoshinaka et al., 2003; Morino et al., 2008). *S. aureus* Mrp is expected to be a target protein of a novel antibiotic because since growth inhibition of *S. aureus* is suppressed by inhibiting translation of the *mrpD* gene using antisense RNA (Ji et al., 2001).

Polyextremophiles such as *Natranaerobius thermophilus* are halophilic, alkaliphilic, and thermophilic bacteria that grow optimally at 3.5 M Na^+^, pH 9.5, and 53°C–55°C (Mesbah et al., 2009). This bacterium has at least eight electrogenic Na^+^(K^+^)/H^+^ antiporters. One of them, Nt-Nha, has homology with MrpA and MrpD, the two large subunits of group 1. In previous studies, none of the Mrp antiporters exhibited antiport activity with MrpA or MrpD alone. However, this Nt-Nha alone showed Na^+^ (K^+^)/H^+^ antiport activity. This supports the suggestion that MrpA and MrpD are critical for the ion transport pathway for antiporters in the CPA 3 family (Krulwich et al., 2009). Recently, study of the Mrp complex of *Methanosarcina acetivorans* from the archaeal domain suggested that MrpA is essential for antiport activity and that the MrpA/MrpD subcomplex is critical for catalyzing Na^+^/H^+^ antiport activity (Jasso-Chavez et al., 2017). This is the second example showing that the Mrp complex exhibits antiport activity even without all its subunits. The consequence of this observation is discussed in Section “Mrp Antiporters from Archaea”.

## Mrp Antiporters from Gram-Negative Bacteria

*Sinorhizobium meliloti* has two sets of *mrp* (*pha*) gene clusters, one belongs to Group 1 (Pha2) and the other belongs to Group 2 (Pha1). The *pha1* gene cluster (SMc03179 to 03184) was identified as a mutation insertion site in a potassium-sensitive strain of the root nodule bacterium *Sinorhizobium meliloti* (Putnoky et al., 1998). *Sinorhizobium*, a symbiotic bacterium, retains potassium-dependent alkaline pH homeostasis ability; however, *pha1* deficiency reportedly causes a loss of alkaline environmental adaptability (Putnoky et al., 1998). Detailed analysis revealed that the *pha1* gene cluster derived from *Sinorhizobium* encodes a K^+^ (Na^+^)/H^+^ antiporter (Putnoky et al., 1998; Yamaguchi et al., 2009).

The *mrp* (*sha*) gene cluster has also been found in *Pseudomonas aeruginosa*, and it reportedly encodes a Na^+^/H^+^ antiporter. Furthermore, inactivation of the *mrp* gene cluster in *P. aeruginos*a PAO1 has been reported to cause reduced pathogenicity (Kosono et al., 2005).

In a study of the group 2 Mrp antiporter of *Vibrio cholerae*, expressed in a major Na^+^/H^+^ antiporter-deficient *E. coli* strain, EP432, this antiporter had Na^+^ (Li^+^, K^+^)/H^+^ antiport activity with optimal pH at pH 9–9.5 and also showed bile acid resistance in *E. coli* (Dzioba-Winogrodzki et al., 2009). A deletion mutant of the group 2 *mrp* gene cluster from *V. cholerae* revealed mutant physiological defects in nitrogen metabolism, cell motility, and biofilm formation (Aagesen et al., 2016).

In a study of the group 1 Mrp antiporter of *Thermomicrobium roseum* expressed in a Na^+^/H^+^ antiporter-deficient *E. coli* strain, KNabc, it was surprisingly found that this antiporter does not catalyze monovalent cation/proton antiport similar to the Mrp antiporters studied to date but catalyzes Ca^2+^/H^+^ antiport in *E. coli* membrane vesicles (Morino and Ito, 2012). This bacterium was isolated from an alkaline hot spring in Yellowstone National Park (Jackson et al., 1973).

The gene cluster of a halotolerant cyanobacterium, *Aphanothece halophytica mrp* (Ah-mrp), which belongs to group 3, has a characteristic genetic structure that retains two *mrpD* genes in an unusual gene order (*mrpCD1D2EFGAB*). Study of a sodium-sensitive mutant *E. coli* expressing Ah-mrp showed that the cyanobacterial Mrp antiporter functions as a Na^+^/H^+^ antiporter and also contributes to sodium tolerance (Fukaya et al., 2009). Another cyanobacterium, *Anabaena* sp. strain PCC 7120, has a group 1 Mrp antiporter. Growth and photosynthesis were inhibited in a *mrpA* mutant cyanobacterial strain (Blanco-Rivero et al., 2009).

It has been reported that the group 1 Mrp antiporters of the halotolerant alkaliphile *Halomonas* sp. Y2 and the halophilic and alkaliphilic *Halomonas zhadongensis* had Na^+^ (Li^+^, K^+^)/H^+^ antiporter functions under alkaline conditions (Meng et al., 2014; Cheng et al., 2016).

## Mrp Antiporters from Archaea

Many Mrp complexes are annotated not only from bacterial genomes but also from archaea (Swartz et al., 2005). The Mrp antiporter from the methanogen *Methanosarcina acetivorans* C2A is composed of a group 1 type of gene cluster comprising seven genes (*mrpABCDEFG*). This Mrp complex plays an essential role in efficient ATP synthesis and optimal growth under conditions with low concentrations of acetic acid in the environment (Jasso-Chavez et al., 2013). Deficiency of a major Na^+^/H^+^ antiporter in *E. coli* cells expressing only MrpA from *M. acetivorans* was still associated with Na^+^/H^+^ antiport activity, although the *K*_m_ value was as low as ca. 50 mM (Jasso-Chavez et al., 2017). The details of these transport mechanisms have not yet been reported.

In hyperthermophilic archaea, Mrp is reported to be involved in the metabolic system of hydrogen production (Kim et al., 2010; Lim et al., 2010, 2014; Schut et al., 2013; Boyd et al., 2014). It is known that a hydrogenase involved in hydrogen production of *Pyrococcus furiosus* and *Thermococcus onnurineus* NA1 is composed of a [NiFe] hydrogenase domain (Mbh) and Mrp type Na^+^/H^+^ antiporter domain. However, there have been no reports of measurement of Mrp antiport activity in these strains/species (Schut et al., 2013). Given the considerable interest in this issue, it is anticipated that the details of the Mrp antiporter that is involved in archaeal energy production will soon be clarified.

## Prediction of the Ion Transport Route in the Mrp Antiporter

**FIGURE 6:**
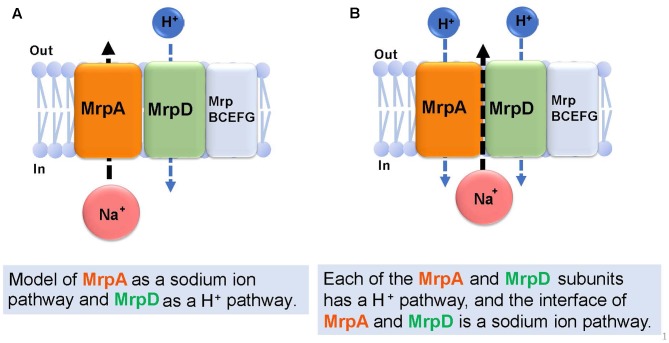
Ion transport pathway model of the Mrp antiporter. Based on previous studies, there are two kinds of ion transport models for the Mrp antiporter. The first model **(A)** proposes MrpA as a Na^+^ pathway and MrpD as a H^+^ pathway (Moparthi et al., 2011). The second model **(B)** proposes that each of the MrpA and MrpD subunits has a H^+^ pathway, and the interface of MrpA and MrpD is a Na^+^ pathway (Sazanov, 2014).

**Figure 6** describes the prediction of the ion transport pathway of the Mrp antiporter (Moparthi and Hägerhäll, 2011; Moparthi et al., 2011; Sazanov, 2015). Owing to homology with the respiratory chain complex I subunit, it is expected that the Mrp antiporter is involved in an ion transport pathway via the MrpA and MrpD subunits. MrpA has the closest homology to the NuoL subunit of complex I and MrpD has the closest homology to complex I NuoM and NuoN subunits. Because the *nuoL*-deficient strain does not transport Na^+^, it was suggested that the NuoL subunit is involved in Na^+^ transport (Marreiros et al., 2014). Moreover, Na^+^ transport was previously demonstrated by the NuoL subunit (Steuber, 2003; Gemperli et al., 2007). Moparthi et al. (2011) reported that the phenotypes of an *mrpA*-deficient strain and *mrpD*-deficient strain of *B. subtilis* are complemented by expressing, respectively, NuoL and NuoN of the respiratory chain complex I of *E. coli*. These observations prompted them to propose that MrpA transports Na^+^, whereas MrpD transports H^+^ in the opposite direction, resulting in antiport activity (Moparthi et al., 2011) (**Figure 6A**).

Sazanov reported that, at the interface between the transmembrane region (TM 5) of the MrpA subunit and the transmembrane region (TM 12) of the MrpD subunit, a Na^+^ transport pathway forms, which was confirmed from a homology model of the MrpA and MrpD subunits constructed from the results of crystal structure analyses of NuoL, NuoM, and NuoN (Sazanov, 2015). This model proposes that highly conserved glutamic acid residues in the NDH-1 motif that is common to the NuoL, NuoM, NuoN, MrpA, and MrpD subunits, function as cation binding sites (**Figure 6B**).

## Prospects for the Future

It is expected that the details of Mrp antiporter complexes and their functional properties as revealed by recent studies will help to reveal the mechanisms of adaptation to environmental conditions not only in alkaliphilic bacteria but also in many other bacteria. The Mrp antiporter plays a major role in the environmental adaptation of a wide variety of bacteria, including pathogenic ones. Furthermore, because Mrp is only found in prokaryotes, studies may lead to the development of inhibitors of the roles of Mrp antiporters that are important in the host.

## Author Contributions

The idea for this review paper was proposed by MI, MM, and TK. The paper was written by MI and TK.

## Conflict of Interest Statement

The authors declare that the research was conducted in the absence of any commercial or financial relationships that could be construed as a potential conflict of interest.
